# Switching Patients With Congenital Adrenal Hyperplasia to Modified‐Release Hydrocortisone Capsules: Relative Bioavailability and Disease Control

**DOI:** 10.1111/cen.15275

**Published:** 2025-05-16

**Authors:** Richard John M. Ross, Wiebke Arlt, Aude Brac de la Perriere, Angelica Lindén Hirschberg, Anders Juul, Deborah P. Merke, John D. C. Newell‐Price, Alessandro Prete, D. Aled Rees, Nicole Reisch, Monica Stikkelbroeck, Philippe A. Touraine, Kerry Maltby, Jo Quirke, Helen Coope, John Porter

**Affiliations:** ^1^ University of Sheffield Sheffield UK; ^2^ MRC Laboratory of Medical Sciences London UK; ^3^ Institute of Clinical Sciences, Imperial College London London UK; ^4^ Hopital Louis Pradel Bron France; ^5^ Karolinska Institutet and Karolinska University Hospital Stockholm Sweden; ^6^ Department of Growth and Reproduction Rigshospitalet Copenhagen Denmark; ^7^ National Institutes of Health Clinical Center and the Eunice Kennedy Shriver National Institute of Child Health and Human Development Bethesda Maryland USA; ^8^ Institute of Metabolism and Systems Research University of Birmingham Birmingham UK; ^9^ Cardiff University Cardiff UK; ^10^ Medizinische Klinik und Poliklinik IV, LMU Klinikum München Munich Germany; ^11^ Radboud Univ Nijmegen Med Ctr Nijmegen Netherlands; ^12^ Hôpital Pitié Salpêtrière Sorbonne Université Paris Cedex France; ^13^ Neurocrine UK Cardiff UK

**Keywords:** 21‐hydroxylase deficiency, adrenal insufficiency, congenital adrenal hyperplasia, glucocorticoid, hydrocortisone, MRHC

## Abstract

**Background:**

Replacement therapy with modified‐release hydrocortisone capsules (MRHC) restores the physiological circadian cortisol rhythm in congenital adrenal hyperplasia (CAH).

**Aims:**

To determine the relative bioavailability of MRHC and evaluate an optimal protocol to switch CAH patients from standard therapy to MRHC.

**Methods:**

(1): Crossover study in healthy participants comparing relative bioavailability of MRHC with immediate‐release hydrocortisone (IRHC). (2): Post hoc analysis of first 4 weeks of phase 3 MRHC study when CAH patients were switched to MRHC.

**Results:**

Twenty‐four healthy male participants completed the relative bioavailability study: 20 mg MRHC showed comparable bioavailability to 20 mg IRHC tablets; mean AUC_0−inf_ was 2650 versus 2450 h*nmol/L, ratio of 108% (90% confidence interval (CI) 103%−113%). In the phase 3 study, 122 CAH patients were recruited of which 63 patients were managed with IRHC alone at baseline; 31 of 63 were randomised to continue IRHC and 32 of 63 were randomised to switch to MRHC on the same daily dose but given twice daily. At 4 weeks, a greater reduction in both the 09:00 h 17‐hydroxyprogesterone and androstenedione was observed in the MRHC group compared to the IRHC group; *p* < 0.001 and *p* = 0.01, respectively.

**Conclusions:**

MRHC showed comparable bioavailability to IRHC based on cortisol AUC after 20 mg administration. Switching patients treated with IRHC to a twice daily MRHC regimen on the same daily dose (giving approximately two thirds of the dose at night) is an effective protocol for starting MRHC treatment.

**Abbreviations**
17OHP: 17‐hydroxyprogesterone
A4: androstenedione
ACTH: adrenocorticotropic hormone
AUC: area under the curve
BMI: body mass index
CAH: congenital adrenal hyperplasia
CHC: chronocort
CI: confidence interval
HC: hydrocortisone
IRHC: immediate‐release hydrocortisone
MRHC: modified‐release hydrocortisone capsules
PK: pharmacokinetic

## 1 Introduction

The goal of treatment in congenital adrenal hyperplasia (CAH) is to control excess adrenal androgen production while using the lowest dose of glucocorticoid that is effective and safe for adrenal replacement [1]. This has been challenging to date because standard immediate‐release glucocorticoid preparations cannot control the overnight rise in adrenocorticotropic hormone (ACTH). This drives excess adrenal androgen production, leading to poor treatment outcomes [2]. To address this, a modified‐release hard capsule formulation of hydrocortisone (MRHC) was developed, with the development name Chronocort, and is now licensed in Europe to treat patients with CAH (Efmody, Neurocrine BV). MRHC is a multi‐particulate formulation in capsules with a modified‐release coating that allows for the delayed and sustained absorption of hydrocortisone [3]. Taken twice daily, at bedtime and upon awakening in the morning, MRHC results in a replication of the overnight rise and diurnal rhythm of cortisol. This improvement in biochemical control of CAH with MRHC has been demonstrated in phase 2 and 3 clinical trials [4, 5]. However, a key question raised by clinicians during MRHC development was defining how to optimally switch patients from standard hydrocortisone therapy to MRHC. To address this question we investigated the relative bioavailability of MRHC in healthy volunteers and analysed adrenal androgen levels in patients switching from immediate‐release hydrocortisone (IRHC) tablets to MRHC in the phase 3 study [5].

## 2 Methods

### 2.1 Relative Bioavailability in Healthy Participants

An open‐label, randomised, 2‐period, crossover study compared the relative bioavailability of MRHC capsules 20 mg (Efmody, Neurocrine BV) with IRHC 20 mg (Cortef, Pfizer, USA) in dexamethasone‐suppressed healthy volunteers. The study was performed in a phase 1 centre at Simbec, Merthyr Tydfil, Wales. Inclusion criteria were healthy men between 18 and 45 years of age, with a body mass index (BMI) of 18−30 kg/m^2^. The primary endpoint was area under the cortisol concentration time curve extrapolated to infinity (AUC_0−inf_), with relative bioavailability considered met if the ratio of cortisol AUC_0−inf_ was between 80% and 125%. All participants gave written informed consent. The study protocols were approved by The Wales Research Ethics Committee (17/WA/0333) and the Medicines and Healthcare Products Regulatory Agency (EudraCT NUMBER:2016 001390 32, NCT03343327). Eligible subjects were randomised to receive a single dose of either MRHC (20 mg) or IRHC tablets (20 mg) over two treatment periods. Dexamethasone (1 mg) was administered at approximately 22:00 h on Day −1 and at approximately 06:00, 12:00, 18:00 and 22:00 h on Day 0 of each study period. Each treatment period was approximately 2 days in duration, from the afternoon before dosing (Day −1) until 24 h post‐dose (Day 1). MRHC and IRHC tablets were administered on the morning (approximately 08:00 h) of Day 0 fasted (after an overnight fast of at least 10 h). Pharmacokinetic (PK) samples for the measurement of serum cortisol were collected at baseline (3 samples at 5‐min (min) intervals starting at −0.5 h before dosing), pre‐dose (0 h) and 23 samples up to 24 h post‐dose (Day 1). Cortisol was measured by HPLC–tandem mass spectrometry (Seirian Laboratories, Merthyr Tydfil). The bioavailability analysis was performed using the industry standard programme WinNonlin (Certara USA). Baseline cortisol suppression was defined as at least two of three baseline serum cortisol samples < 50 nmol/L (<18 ng/mL) and the average of the three baseline results < 50 nmol/L at both treatment periods.

### 2.2 Switch Protocol in CAH Patients

In the phase 3 MRHC study, 63 CAH patients were taking IRHC tablets at baseline, of which 31 were randomised to continue on hydrocortisone tablets and 32 were randomised to switch to the same daily dose of MRHC, given approximately two thirds of the daily dose at bedtime and one‐third of the daily dose upon awakening in the morning. At baseline, the relative doses for MRHC and IRHC were 20 and 23.75 mg, respectively. No dose titration or dose changes were performed for the patients during the 4 week study period with the exception of dose changes due to safety reasons. Serum 17‐hydroxyprogesterone (17OHP) and androstenedione (A4) were measured at 09:00 h at baseline and 4 weeks by HPLC–tandem mass spectrometry (Q2 Solutions, USA). The key inclusion criterion was a diagnosis of classic 21‐hydroxylase deficient CAH. All participants gave written informed consent. The study protocols were approved by The East Midlands—Leicester Central Research Ethics Committee (ref: 16/EM/0278) and the Medicines and Healthcare Products Regulatory Agency (NCT03062280; Eudract 2015‐005448‐32). Data are presented as geomean and statistical comparison of baseline and 4‐week data was made by Mann−Whitney 2‐tailed test.

## 3 Results

### 3.1 Bioavailability Study (Table 1 and Figure 1)

**Table 1:** Cortisol pharmacokinetic (PK) parameters (baseline adjusted) after intake of modified‐release hydrocortisone (MRHC) in comparison to immediate‐release hydrocortisone (IRHC) in healthy male participants.

PK Parameter	20 mg MRHC	20 mg Cortef
C_max_ (nmol/L) Maximum concentration (*n* = 24)	609	788
T_max_ (h) Time of maximum concentration (*n* = 24)	5.00	1.00
AUC_0−inf_ (h^a^ nmol/L) (*n* = 24)	2650	2460^a^
k_el_ (1/h) The elimination rate constant (*n* = 24)	0.466	0.483
t_½_ (h) serum half‐life (*n* = 24)	1.49	1.43
CL/F (L/h) Clearance (*n* = 24)	20.8	22.5
V_z_/F (L) Apparent volume of distribution (*n* = 24)	44.7	46.5

*Note:* Presented as geomean except for Tmax that is median.

a Based on 21 subjects only, for subjects 001, 014 and 018 no values were calculated.

**Figure 1:**
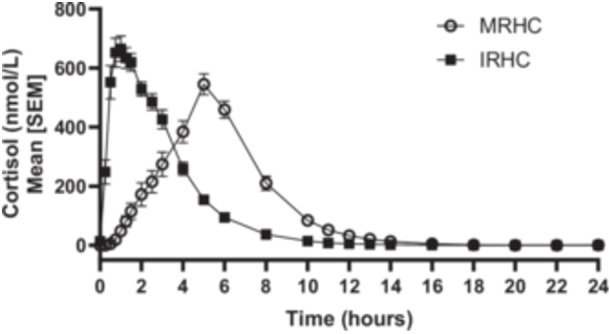
Baseline corrected cortisol profiles in 24 dexamethasone suppressed healthy males after oral intake of 20 mg modified‐release hydrocortisone capsules (MRHC) and immediate‐release hydrocortisone tablets (IRHC), respectively.

Twenty‐five healthy male subjects were randomised, with one subject excluded because they tested positive for a banned substance. Of the 24 subjects who completed the relative bioavailability study, three were excluded from the primary end point analysis: two were excluded because their baseline cortisol was not suppressed and one because there were insufficient data to calculate the AUC. The primary end‐point showed comparable relative bioavailability between 20 mg MRHC capsules and 20 mg reference hydrocortisone: Mean AUC_0−inf_ was 2650 versus 2450 h*nmol/L, ratio of 108% (90% confidence interval [CI] 103%−113%). There were no severe or serious treatment emergent adverse events (TEAEs) or suspected unexpected serious adverse reactions (SUSARs) reported.

### 3.2 Switching Patients to MRHC (Figure 2)

**Figure 2:**
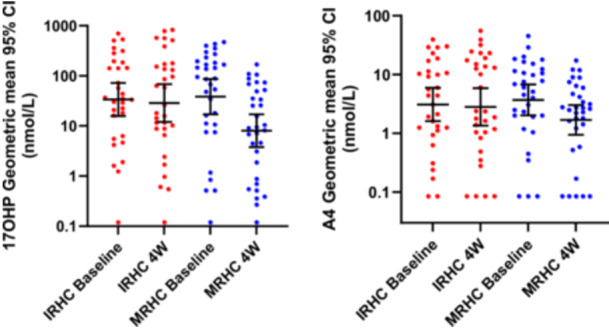
09:00 h serum 17OHP and A4 at baseline all on immediate‐release hydrocortisone tablets (IRHC) and after 4 weeks treatment with either modified‐release hydrocortisone capsules (MRHC) (*n* = 32) or IRHC (*n* = 31).

In the phase 3 MRHC study 63 patients were taking IRHC alone at baseline; of these, 31 were randomised to continue on IRHC and 32 were randomised to switch to MRHC. They were assessed by the measurement of 09:00 h 17OHP and A4 at baseline and 4 weeks. At baseline there was no difference between the MRHC and IRHC groups for either geomean 17OHP, 38.6 versus 33.7 nmol/L or A4, 3.7 versus 3.1 nmol/L. At 4 weeks, 17OHP and A4 had fallen significantly in the MRHC group compared to the IRHC group, with the median change for 17OHP at −52.1 versus +7 nmol/L (*p* = 0.0003) and geomeans of 8.0 versus 28.7 nmol/L (*p* = 0.0196), and for A4 at −1.5 versus +0.1 nmol/L (*p* = 0.0031) with geomeans of 1.7 versus 2.8 nmol/L (*p* = NS). During the 4‐week period, there were no adrenal crises in either group, and there were nine episodes of sick‐day stress dosing with MRHC versus eight with IRHC.

## 4 Discussion

In this crossover study, MRHC demonstrated comparable relative bioavailability to IRHC. Switching patients with CAH to MRHC from IRHC at the same hydrocortisone dose (20 mg) resulted in improved biochemical control of CAH. Episodes of sick day stress dosing between the groups were similar, with no adrenal crises. These are important observations for clinicians to consider, as modifying mechanisms of drug release can affect bioavailability.

It is important to note that the assessment of the bioavailability of oral hydrocortisone is complicated by its saturable binding in the therapeutic range to cortisol‐binding globulin. Ninety percent of serum cortisol circulates bound to cortisol‐binding globulin, 5% to generic binding proteins, such as albumin and α−1 glycoprotein, and only 5% is unbound or ‘free’ [6]. When corrected for saturable binding, IRHC is completely absorbed after oral administration with the mean (95% CI) bioavailability of oral hydrocortisone calculated from serum cortisol of 1.00 (0.89−1.14) [7]. Additionally, modifying the release of hydrocortisone has been previously associated with reduced bioavailability. A previous modified‐release tablet formulation of hydrocortisone, brand name Plenadren, showed a 20% reduction in bioavailability [8], and when developing modified hydrocortisone formulations, we found that adding a sustained release layer resulted in reduced bioavailability [3]. The unique final formulation of MRHC has a delayed release coating, and the results of the study reported here show it has comparable bioavailability to IRHC based on the cortisol AUC (mean AUC_0−inf_ was 2650 vs. 2450 h*nmol/L, ratio of 108% with 90% CI 103%−113%). As would be predicted, the Cmax for MRHC was lower than that for IRHC and this has the advantage that MRHC maintains cortisol levels within the physiological range without exceeding the protein binding capacity. The Tmax for MRHC in this study was shorter (5 h) compared to that previously reported (8 h) for twice daily MRHC [3]. The reason for this was that in the current bioavailability study, MRHC was given on a fasted stomach in the morning, whereas in the previous study it was given at night and the slower gut transit at this time delays MRHC reaching the small bowel where the pH resistant coat is triggered. Clearance of cortisol was similar for MRHC and IRHC, and cortisol levels fall to low levels in the evening on MRHC as occurs physiologically [3].

The biochemical monitoring of CAH in patients receiving IRHC usually uses morning serum 17OHP and A4 with the aim of keeping the 17OHP below 3‐4 times the upper limit of normal and the A4 below the upper limit of normal for the reference range [1]. Standard glucocorticoid replacement cannot control CAH throughout the 24 h so, some clinicians use hormone profiles sampling throughout the 24 h to monitor control. An advantage of MRHC is that because it replaces the cortisol circadian rhythm a sample taken in the morning or afternoon reflects over all control. It is recognised that on standard hydrocortisone regimens, a higher daily dose of glucocorticoid is required to achieve biochemical control than that required for adrenal replacement, which was the rationale for the development of MRHC. It can be seen from the data presented here that 17OHP and A4 levels vary widely in CAH patients. Importantly, MRHC brought the 17OHP and A4 levels down more effectively than the same dose of IRHC, giving the potential to reduce the daily dose of glucocorticoid as was seen in the MRHC phase three studies [5].

MRHC is designed to replace the overnight rise in cortisol and thereby replace the diurnal rhythm of cortisol. The dose, when given last thing at night, does not start releasing cortisol until the early morning, and peaks around the time of waking. In the phase 3 study, approximately two‐thirds of the daily dose was given at bedtime and one‐third given upon awakening in the morning. The results from this study suggest that when switching patients to MRHC, this is an effective regimen, allowing for androgen control without increasing the risk of stress dosing episodes. There were no new safety signals using this switching regimen. As a practical example, a patient on 25 mg of IRHC could be switched to MRHC 15 mg at night, and 10 mg given first thing in the morning. For 20 mg IRHC, this could be switched to 15 mg of MRHC at night and 5 mg of MRHC in the morning; and for 15 mg IRHC, this could be 10 mg of MRHC at night and 5 mg of MRHC in the morning. For stress dosing, patients should use IRHC.

In conclusion, switching patients from standard IRHC therapy to modified‐release MRHC twice daily can be done by using the same total daily dose and delivering this with the MRHC formulation: approximately two‐thirds of the daily dose at night and one‐third in the morning. It can be expected that the biochemical control of CAH will improve, and, in time, it may be possible to reduce the total daily dose of hydrocortisone.

## Conflicts of Interest

The authors have the following conflicts of interest to declare in relation to this study: W.A., A.B.P., A.L.H., A.J, D.P.M, J.N.P., A.P., D.A.R., N.R., & N.S. were study investigators. D.P.M has received research funds from Diurnal Ltd (now Neurocrine UK Limited) through an NIH Cooperative Research and Development Agreement. R.J.R. and J.P. are consultants to, and H.C., K.M. and J.Q. are employees of Neurocrine UK Limited. D.P.M. received research funds from Diurnal Limited (now Neurocrine UK Limited), Neurocrine Biosciences and Adrenas Therapeutics through the National Institutes of Health Cooperative Research and Development Agreements.
